# Comparative ^1^H NMR Metabolomics Between Scandinavian Propolis and Australian Propolis: The Quest to Identify Radical Scavenging Compounds

**DOI:** 10.1002/mrc.70082

**Published:** 2026-01-28

**Authors:** Jonas Vind, Søren Balling Engelsen, Henrik Munch Jørgensen, Julie Christine Antvorskov, Knud Josefsen, Violetta Aru

**Affiliations:** ^1^ Department of Pathology, The Bartholin Institute Copenhagen University Hospital Copenhagen Denmark; ^2^ Department of Food Science University of Copenhagen Frederiksberg Denmark; ^3^ Department of Clinical Research Steno Diabetes Center Copenhagen, Translational Type 1 Diabetes Research Herlev Denmark

**Keywords:** ^1^H NMR metabolomics, *A. mellifera*, cerumen, ferulic acid, *p*‐coumaric acid, propolis, radical scavenging activity (RSA), *r*PLS, STOCSY, *T. carbonaria*

## Abstract

Propolis from *Apis mellifera* and cerumen from *Tetragonula carbonaria* are complex mixtures of beeswax, plant resins, and bee secretions whose composition varies with geography and species. Understanding these differences is important for exploring their bioactive potential. This study employs untargeted quantitative ^1^H NMR metabolomics to characterize *A. mellifera* propolis from Scandinavia (Denmark and Norway) and Australia, as well as cerumen from *T. carbonaria* in Australia. Hydrophilic and hydrophobic extracts were analyzed to assess compositional differences across geographical origin and bee species, and to link specific metabolites to radical scavenging activity (RSA). Principal component analysis (PCA) of the ^1^H NMR spectra showed a marked separation between Scandinavian and Australian propolis. Hydrophilic extracts showed that Scandinavian propolis contains higher levels of aromatic compounds, whereas Australian propolis is richer in carbohydrates. In contrast, cerumen from *T. carbonaria* exhibits higher amounts of terpenoids. Hydrophobic extracts revealed that Australian propolis has the highest wax content, with shorter chains and more free fatty acids, while Scandinavian propolis samples display uniform wax structures and the highest aromatic content. Multivariate regression using recursive weighted partial least squares (*r*PLS) to RSA prediction highlighted signals attributable to ferulic acid and *p*‐coumaric acid, which were confirmed by statistical total correlation spectroscopy (STOCSY). These findings demonstrate the utility of quantitative ^1^H NMR metabolomics for distinguishing botanical and geographic chemotypes of propolis and cerumen. The findings further show that Scandinavian propolis is more consistent with respect to metabolite composition compared to Australian samples, presumably reflecting differences in resin sources for foraging.

## Graphical abstract

Propolis from Scandinavia and Australia was chemically characterized using ^1^H NMR spectroscopy and multivariate modeling. Recursive partial least squares revealed spectral features associated with radical scavenging activity, and STOCSY enabled identification of the key phenolic compounds underlying these bioactive features.

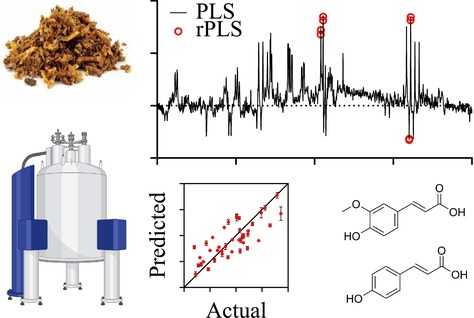

## 1 Introduction

Propolis is a wax‐containing resinous material produced by honeybees ( *Apis mellifera* ) [1] and is used to fill crevices, reinforce hive structures, and protect against pathogens [2]. Due to its well‐established antioxidative properties, propolis is used as a nutraceutical [3]. Clinical studies have reported that propolis supplementation can modulate endogenous redox‐related biomarkers, including increasing reduced glutathione [4] and glutathione peroxidase [5] levels. Although the anti‐oxidative properties are often linked to polyphenols [6], the specific origin of these antioxidative effects remains elusive [7].

Stingless bees also produce a similar substance known as cerumen, which plays a crucial role in constructing internal nest structures such as brood cells and honeypots, while also serving as a physical and chemical defense [8, 9]. Although *A. mellifera* has become globally widespread due to human domestication [10], stingless bees, such as *Tetragonula carbonaria* , remain naturally restricted to tropical and subtropical climates. Their limited ability to thermoregulate shapes both their nesting behavior and beekeeping potential [11]. As a result, despite the existence of over 500 stingless bee species [12], research on cerumen remains limited, and its chemical composition and bioactive potential are not yet fully understood to the extent of propolis.

Both propolis and cerumen are primarily composed of beeswax, foraged resins, and enzyme‐rich bee secretions [13, 14]. While beeswax mainly consists of saturated hydrocarbons together with their ester and acid derivatives [15, 16], resins are chemically complex and contain a wide range of bioactive compounds, including flavonoids, phenolic acids, and terpenoids [17, 18]. The composition of foraged resins varies depending on geography, local flora, and bee species, reflecting hive‐ and nest‐specific functional demands and contributing significantly to the chemical diversity of propolis and cerumen [19, 20].

Advances in analytical techniques have enabled detailed investigations of propolis, and in recent years increasing efforts have focused on comparative chemical characterization and bioactivity of both propolis and cerumen across geographic regions and bee species [21, 22, 23]. Studies consistently identify phenolic acids and flavonoids as dominant constituents of *A. mellifera* propolis, with regional differences shaping both composition and bioactivity [24, 25]. Egyptian propolis has even been shown to act synergistically with honey against multidrug‐resistant uropathogens [26]. In contrast, cerumen from stingless bees contains polyphenols but is particularly enriched in terpenoids [9, 21]. Spectroscopic and chromatographic approaches have further identified unique chemical markers that distinguish different chemotypes and provide insight into the resin sources used by bees [27, 28]. Collectively, these findings highlight that propolis and cerumen are complex yet chemically traceable matrices.

Proton nuclear magnetic resonance (^1^H NMR) spectroscopy provides a high‐resolution, untargeted approach for profiling compounds in complex samples such as foods and natural products [29, 30]. ^1^H NMR delivers a comprehensive and inherently quantitative view of chemical composition, making it particularly well‐suited for exploratory comparative studies of propolis and cerumen [31]. When combined with multivariate data analysis techniques [30, 32], ^1^H NMR offers a robust and widely adopted platform for untargeted metabolomics of low‐molecular‐weight compounds (< 1.5 kDa) in biological systems [30, 33]. In this context, metabolomics can translate complex spectral data into interpretable biochemical signatures, enabling robust classification, biomarker discovery, and quality control across diverse samples [34]. To date, however, ^1^H NMR metabolomics studies of propolis and cerumen have largely focused on compositional profiling and geographical discrimination, with fewer studies investigating their biological activity or linking specific metabolites to functional effects [35].

In this study, ^1^H NMR metabolomics was employed to investigate how bee species ( *A. mellifera* and *T. carbonaria* ) and geographical origin (Australia, Denmark, and Norway) influence the chemical composition of propolis and cerumen, considering both resin‐derived constituents and beeswax. Propolis and cerumen extracts were measured by ^1^H NMR spectroscopy. To identify candidate molecules contributing to the antioxidative capacity measured by the 2,2‐diphenyl‐1‐picrylhydrazyl (DPPH) radical scavenging activity (RSA) assay [36], recursive weighted partial least squares regression (*r*PLS) [37] and statistical total correlation spectroscopy (STOCSY) were employed [38]. The combination of the two approaches enabled the identification of signals from compounds of interest and the detection of interrelated spectral features, thereby facilitating compound assignment of putative metabolite candidates responsible for the RSA activity.

## 2 Experimental Section

### 2.1 Chemicals

Analytical grade methanol (CH_3_OH, P ≥ X%), chloroform (CHCl_3_, P ≥ X%), deuterium oxide (D_2_O, 99.9 atom % D), tetramethylsilane (TMS, ≥ 99.9%), 3‐(trimethylsilyl)propionic‐2,2,3,3‐d4 acid sodium salt (TSP‐d4, 98 atom % D, P ≥ 98.0%), potassium phosphate (KH_2_PO_4_, P ≥ 99.0%), dibasic potassium phosphate (K_2_HPO_4_, P ≥ 98.0%), and sodium azide (NaN_3_, P ≥ 99.5%) and high‐performance liquid chromatography (HPLC) grade *p*‐coumaric acid (≥ 98.0%) and ferulic acid (99%) were purchased from Sigma‐Aldrich (Merk KGaA, Darmstadt, Germany). MilliQ water was obtained using a Millipore lab water system (Merck KGaA) equipped with a 0.22‐μm membrane filter.

### 2.2 Sample Collection

Propolis ( *A. mellifera* ) and cerumen ( *T. carbonaria* ) samples were obtained from local beekeepers in New South Wales, Australia, across Denmark, and southern Norway (Table S1). Upon collection, the samples were stored at 4°C in food‐grade ziplock bags filled with nitrogen, which were then placed inside airtight, nitrogen‐filled glass containers. In total, 46 propolis samples were analyzed, including 5 from Australia, 35 from Denmark, and 6 from Norway. Three cerumen samples from New South Wales were also analyzed.

### 2.3 Sample Extraction and Preparation for ^1^H NMR Analysis

**FIGURE 1:**
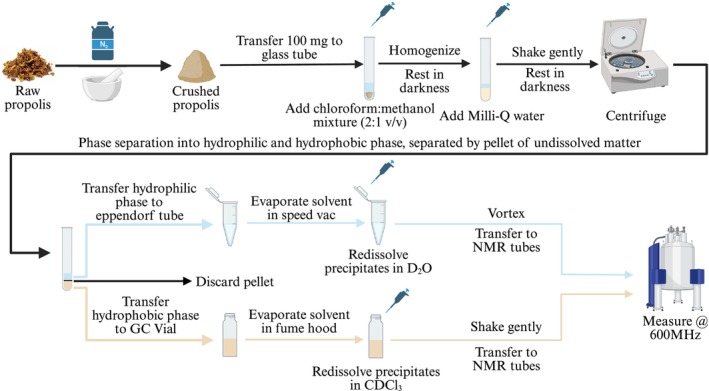
Schematic of the analytical workflow applied to all propolis (*n* = 46) and cerumen (*n* = 3) samples. Each extraction yielded a hydrophilic phase and a hydrophobic phase. All samples were extracted in triplicates.

Raw propolis and cerumen samples were snap‐frozen using liquid nitrogen and subsequently ground into a powder using a pestle and mortar. Extraction was performed according to a modified version of the Folch protocol [39]. Briefly, a 2:1 (v/v) solution containing CHCl_3_ and CH_3_OH was prepared. For each sample, an aliquot of 4 mL of the CHCl_3_‐CH_3_OH solution was added to 100 ± 4 mg of powdered propolis/cerumen in round‐bottom glass tubes and homogenized for 30 s using a T10 Basic ULTRA‐TURRAX (IKA‐Werke, Germany). After an hour of storage in the dark at room temperature, 1 mL of Milli‐Q water was added to each sample. The CHCl_3_‐CH_3_OH‐H_2_O mixture was further mixed by gently tilting the tubes and stored at room temperature in the darkness for an additional hour. At the end of the extraction procedure, samples were centrifuged at 3600 rpm for 15 min at 4°C (Labogene Scanspeed 1580R, Allerød, Denmark). This resulted in the separation of the samples into two phases, a hydrophilic phase on top and a hydrophobic phase at the bottom, divided by a pellet of undissolved matter. For each sample, an aliquot of 1 mL of the top hydrophilic phase was transferred into an Eppendorf tube and dried overnight at 25°C and 1000 rpm using a Scanvac centrifugal vacuum concentrator (LaboGene ApS, Lillerød, Denmark). The dried hydrophilic samples were dissolved in 1 mL of phosphate buffer (pH = 7.6) containing 20% of D_2_O and TSP (5 mM). The mixture was vortexed (Buch and Holm, Scientific Industries Vortex‐Genie 2) at a medium speed to avoid foaming for 5 min. Subsequently, 600 μL of the mixture was transferred into SampleJet NMR tubes (Bruker BioSpin, Ettlingen, Germany) of L = 103.5 mm and O.D. = 5.0 mm and capped with a matching lid. For the bottom hydrophobic phase, 1 mL was transferred into a 1.5‐mL glass vial. The chloroform mixture was dried overnight at room temperature under a fume hood, and the dried sample re‐dissolved in 1.0 mL of CDCl_3_. For each sample, an aliquot of 600 μL was transferred into a SampleJet NMR tube and capped with a matching lid. All samples were extracted in triplicates and stored inside the SampleJet at 4°C until analysis. An overview of the analytical workflow is given in Figure 1.

### 2.4 Standard Addition

For the standard addition experiments, 10mM solutions of ferulic acid and *p*‐coumaric acid were prepared in the before‐mentioned buffer (see Section 2.3). An aliquot of 20 μL was added directly to the NMR tube containing the hydrophilic extract from propolis.

### 2.5 ^1^H NMR Measurements

Samples were measured using a Bruker Avance III operating at a proton Larmor's frequency of 600.13 MHz and equipped with a 5‐mm broadband inverse (BBI) probe (Bruker Biospin, Rheinstetten, Germany). The magnet was equipped with an automated sample changer (SampleJet, Bruker Biospin, Rheinstetten, Germany) with a refrigerated storage station (278 K) and heating/drying station (306 K). Cooling of the probe and SampleJet system were controlled by the BCU (Bruker Cooling Unit). Data acquisition and processing were carried out in the TopSpin software (version 3.6, Bruker, Rheinstetten, Germany). Automation of the overall measurement procedure was controlled by the iconNMR software (Bruker Biospin, Rheinstetten, Germany). The hydrophilic extracts and the samples spiked with standards were measured as described by Forsberg et al. [40]. Briefly, ^1^HNMR spectra were acquired at 300 K using the *noesygppr1d* pulse sequence from the Bruker pulse program library. Each spectrum was collected with 32 scans following four dummy scans, with free induction decays (FIDs) recorded into 65,536 data points across a spectral width of 20 ppm. The acquisition time (AQ), relaxation delay (D1), and mixing time were set to 2.72, 4.0, and 0.01 s, respectively. The receiver gain (RG) was automatically set to of 90.5 for all samples, as described by Forsberg et al. [40]. Following Fourier transform (FT), automatic phasing and baseline correction were performed in the TopSpin software (version), with exponential line broadening applied (LB = 0.3 Hz) applied. An artificial signal at 12 ppm, corresponding to a known concentration of 10 mM protons, was used for data normalization [40]. ^1^H NMR spectra of the hydrophobic extracts were recorded at 300 K, using the *zg* pulse sequence (Bruker pulse program library). A total of 128 scans were acquired following two dummy scans, with FIDs collected into 72,114 data points over a spectral width of 20 ppm. The AQ and D1 were set to 3.0 and 6.0 s, respectively. For each sample, the RG value was automatically determined using the *rga* command (Bruker command nomenclature). As before, data processing, including FT, automatic phasing, and baseline correction, was conducted using TopSpin software.

### 2.6 Data Analysis

#### 2.6.1 Principal Component Analysis (PCA)

The ^1^H NMR spectra were imported into MATLAB R2024a (Mathworks Inc., Natick, MA, USA) where the spectra were referenced to the TMS or TSP singlet (hydrophobic or hydrophilic extract, respectively) at 0.00 ppm and aligned using *i*coshift [41]. Noisy regions, including signals from residual chloroform (hydrophobic extract) and water (hydrophilic extract) at 7.26 and 4.70 ppm, respectively, and the TMS/TSP singlet at 0.00 ppm were removed prior to data analysis. The ^1^H NMR spectra of the hydrophilic extract were normalized to the area of the artificial signal at 12 ppm, while the ^1^H NMR spectra of the hydrophobic extract were normalized to the RG. All spectra were also normalized relative to the mass (mg) of the sample. Principal component analysis (PCA) [42] was performed on the Pareto‐scaled ^1^H‐NMR data from the hydrophilic and hydrophobic propolis extracts, respectively (*n* = 3·46).

#### 2.6.2 Variable Selection by rPLS

An *r*PLS regression approach [37] was employed to identify spectral variables (metabolites) in the ^1^H NMR spectra of the hydrophilic and hydrophobic extracts associated with RSA. Technical replicates were averaged after careful alignment and prior to data modelling, to obtain a single spectrum per sample. Before rPLS modelling, the ^1^H NMR spectra were limited to include only the aromatic region (6.0–8.0 ppm), smoothed using a moving average filter (window size = 5), and Pareto‐scaled. The response variable (RSA) was mean‐centered. Recursive weighting was performed over 25 iterations without active variable pruning. In each iteration, PLS regression was applied with three latent variables (LVs); the absolute regression coefficients were normalized, and cumulative multiplication was used to update variable weights. The predictor matrix was then re‐weighted for the next iteration. Model performance at each iteration was assessed by five‐fold Venetian‐blind cross‐validation (CV), based on the root mean square error of cross‐validation (RMSE_CV_). For comparison, a standard PLS regression model was constructed on the same spectral region and evaluated using identical preprocessing and CV settings.

To assess the stability and predictive performance of the *r*PLS approach, Monte Carlo cross‐validation (MCCV; 1000 repeats) was performed with random 80/20 splits into a training set and an external hold‐out test set. Within each repeat, a five‐fold Venetian‐blind CV was applied to the training set, after which models were refitted on the complete training set to generate predictions for the hold‐out test set. RMSE distributions were collected for both the internal CV and the external test predictions.

#### 2.6.3 Metabolites Assignment

Metabolite assignment was performed using literature data [43], ChemDraw software (Version 23; Revvity Signals Software Inc., Waltham, MA, USA), and previously recorded Infrared (IR) data [44]. Statistical total correlation spectroscopy (STOCSY) analysis [38] was subsequently used to identify signals originating from compounds corresponding to signals highlighted by *r*PLS. The coefficient of determination (*r* ^2^) was computed across the spectra to assess the strength of association. All analyses were conducted in MATLAB R2024a. Identification of ferulic acid and *p*‐coumaric acid was additionally confirmed by standard addition (see Section 2.4). Furthermore, signals originating from beeswax were also identified using STOCSY. Here, the strong singlet at 1.22 ppm, arising from aliphatic methylene of the beeswax, was used as the driver signal. For these analyses, Danish and Norwegian propolis samples were clustered together due to their chemical similarity.

#### 2.6.4 Quantification of Ferulic Acid and *p*‐Coumaric Acid

Quantification of ferulic acid and *p*‐coumaric acid was based on the integral of the most shielded peak in the trans‐olefinic doublet (coupling constant ~16 Hz) characteristic of hydroxycinnamic acids. This analysis was performed exclusively on the hydrophilic extracts, and concentrations were determined from the increase in peak intensity observed in spiking experiments. For these analyses, Danish and Norwegian propolis samples were clustered together due to their chemical similarity.

## 3 Results

### 3.1 ^1^H NMR Spectra of Propolis and Cerumen

**FIGURE 2:**
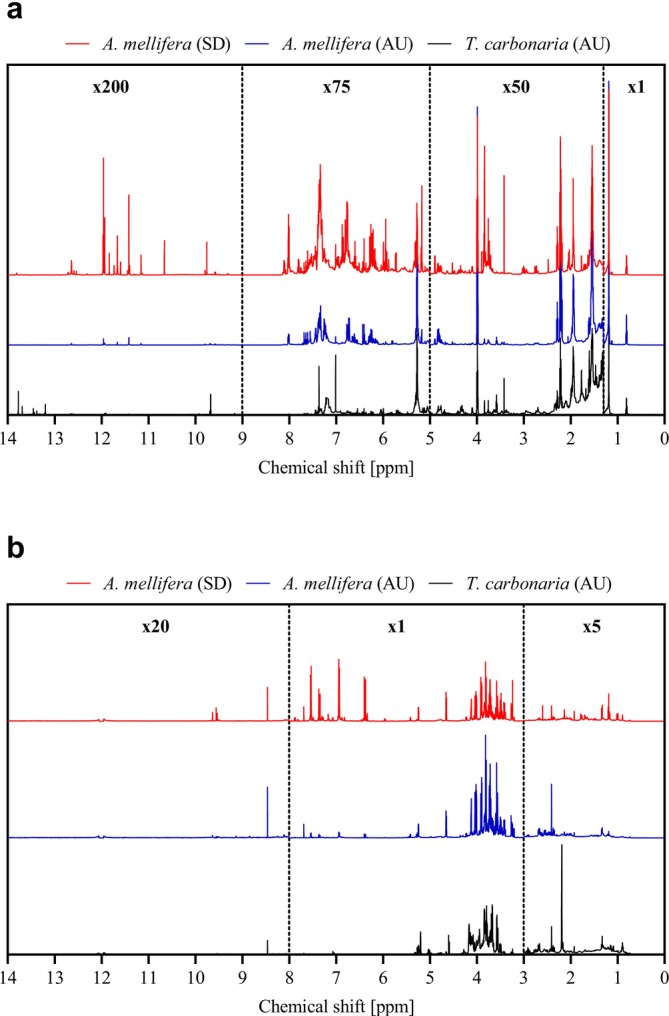
Average ^1^H NMR spectra of propolis extracts from *A. mellifera* in Scandinavia (SC, top spectrum) and Australia (AU, middle spectrum) and cerumen extracts from *T. carbonaria* in Australia (bottom spectrum). ^1^H NMR spectra are shown for the (a) hydrophobic phase and (b) hydrophilic phase, obtained through a modified Folch extraction.

Figure 2 shows the averaged ^1^H NMR profiles of hydrophilic (methanol/water) and hydrophobic (chloroform) fractions of *A. mellifera* propolis collected in Scandinavia and Australia (*n* = 46) and of *T. carbonaria* cerumen collected in Australia (*n* = 3).

In the spectra of the hydrophobic extract (Figure 2a), characteristic beeswax signals were detected across all groups, including a strong singlet at 1.22 ppm (aliphatic methylene, ‐CH_2_‐) and a triplet at 0.88 ppm (J = 7.16 Hz; aliphatic methyl, ‐CH_3_). Beyond these common features, notable differences were apparent in the aromatic and aliphatic regions. Scandinavian propolis exhibited the strongest aromatic signals (6.0–8.0 ppm), followed by Australian propolis, which still displayed more pronounced aromatic peaks than cerumen. In contrast, cerumen was distinguished by a higher density of aliphatic signals (0.5–3.0 ppm) compared to Australian propolis, possibly reflecting terpenoid‐like constituents. However, this interpretation remains tentative and will be discussed later.

The ^1^H NMR spectra of the hydrophilic extract spectra also revealed clear differences between groups (Figure 2b). Propolis samples showed increased signal intensities in the 3.0–4.0 and 4.5–5.5 ppm regions, relative to cerumen. These regions are typically associated with protons adjacent to electronegative atoms, arising from carbohydrates, with the 4.5–5.5 ppm range specifically characteristic of anomeric protons from free sugars and glycosidic moieties. As in the hydrophobic extracts, Scandinavian propolis contained higher concentrations of aromatic compounds than Australian propolis. While Australian propolis displayed very low aromatic content in the hydrophilic extracts, cerumen spectra were virtually devoid of aromatic proton signals.

### 3.2 Wax Analysis

**FIGURE 3:**
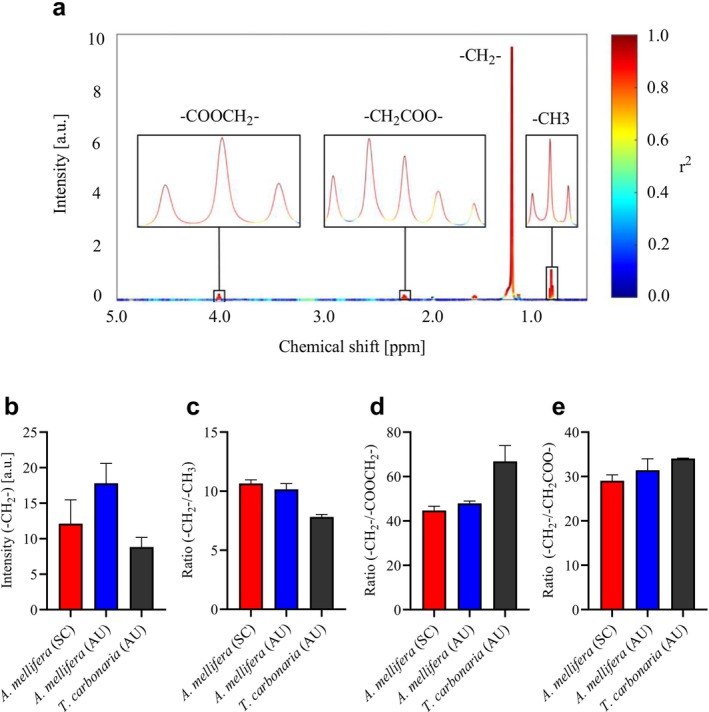
Beeswax content and structural features in propolis from Scandinavia (SC, *n* = 41) and Australia (AU, *n* = 5) *A. mellifera* and cerumen (*n* = 3) from *T. carbonaria* across origin and species. (a) Signals associated with beeswax, highlighted by STOCSY, using the singlet at 1.22 ppm as driver. (b) Total beeswax content estimated from intensity of singlet at 1.22 ppm. (c) Average chain length calculated as the ‐CH_2_‐/‐CH_3_ ratio. (d) Degree of esterification derived from the ‐CH_2_‐/‐COOCH_2_‐ ratio based on the ester‐specific signal at 3.99 ppm. (e) ‐CH_2_‐/‐CH_2_COO‐ ratio based on multiplet at 2.21 ppm. Bars and vertical lines indicate mean + standard deviation (SD).

As beeswax constitutes a major portion of propolis, quantifying wax‐related signals and evaluating structural characteristics provides useful insight into species‐ and region‐specific differences in chemical composition. To this end, STOCSY was applied to identify signals associated with beeswax (Figure 3a). Several signals were highlighted, including resonances at 1.22 ppm (s) and 0.88 ppm (t, J = 7.16 Hz), corresponding to methylene (‐CH_2_‐) and methyl (‐CH_3_) groups, respectively. Additional signals were observed at 2.21 ppm (m) and 3.99 ppm (t, J = 6.68 Hz). The triplet at 3.99 ppm arises from methylene protons positioned alpha to an oxygen atom in esters (COOCH_2_‐), while the multiplet at 2.21 ppm likely originates from methylene protons positioned alpha to carbonyl groups in both esters and carboxylic acids (‐CH_2_COO‐).

These signals were used to assess total beeswax content, average chain length (‐CH_2_‐/‐CH_3_), and esterification (‐CH_2_‐/‐COOCH_2_‐). The ratio of ‐CH_2_‐ to carbonyl‐associated methylene protons (‐CH_2_‐/‐CH_2_COO‐) at 2.21 ppm, arising from both esters and carboxylic acids, was additionally used to evaluate differences potentially attributable to variations in free fatty acid content across origin and species (Figure 3b–e). Propolis from Australia contained higher beeswax levels compared to Scandinavian samples and cerumen. Furthermore, beeswax from Scandinavian and Australian *A. mellifera* showed similar average chain lengths of 10.7 and 10.2 ‐CH_2_‐/‐CH_3_, respectively, whereas *T. carbonaria* wax exhibited shorter chains, averaging 7.8 ‐CH_2_‐/‐CH_3_.

Differences in esterification were minor between *A. mellifera* samples from Scandinavia and Australia, with average ‐CH_2_‐/‐COOCH_2_‐ ratios of 44.7 and 47.9, respectively. In contrast, *T. carbonaria* wax displayed a higher ratio of 66.8, reflecting lower ester content relative to chain backbone. Analysis of the ‐CH_2_‐/‐CH_2_COO‐ ratio showed noticeably smaller differences across species, indicating a higher proportion of free fatty acids in *T. carbonaria* wax compared to *A. mellifera* , whose wax was more extensively esterified. No correlations were observed with highly deshielded proton signals (> 10 ppm) in the STOCSY analysis, consistent with the absence or broadening of carboxylic acid protons due to rapid exchange.

Interpretation of the 2.21 ppm multiplet is complicated by overlapping contributions from fatty esters and free fatty acids. To support the assignment of these components, the ^1^H NMR observations were compared with infrared (IR) spectra of the same samples obtained in a separate study [44] (Figures S1 and S2). The IR data show distinct carbonyl absorptions for esters (1736 cm^−1^) and carboxylic acids (1710 cm^−1^) [44]. *A. mellifera* samples displayed a dominant ester band at 1736 cm^−1^, consistent with an ester‐driven 2.21 ppm multiplet in the NMR, likely reflecting high diester content. In contrast, *T. carbonaria* samples exhibited a stronger 1710 cm^−1^ band accompanied by a broader, more complex NMR multiplet, indicating a greater contribution from free fatty acids. Due to the limited number of *T. carbonaria* samples (*n* = 3), separate statistical correlation was not feasible, and the interpretation remains qualitative. Nonetheless, the combined IR‐NMR evidence indicates a higher free acid content in *T. carbonaria* wax compared with the more esterified wax profile of *A. mellifera* . Supporting IR data are provided in Supporting Information (Figures S1 and S2).

### 3.3 PCA

**FIGURE 4:**
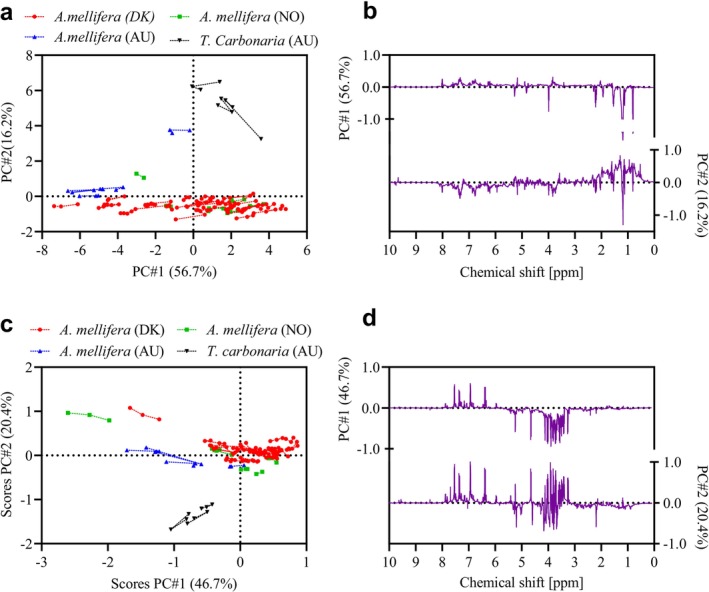
PCA scores and loading plots of ^1^H NMR data from propolis (*n* = 3·46) and cerumen (*n* = 3·3) extracts. (a) Scores and (b) loadings plot for the hydrophobic phase; (c) scores and (d) loadings plot for the hydrophilic phase. Scores are shown with connecting lines for triplicate samples.

PCA was performed on the spectral ^1^H NMR data to investigate the variation among samples based on geographical origin and bee species. A clear species‐ and region‐specific separation can be observed in the scores plot of the hydrophobic extract (Figure 4a). Cerumen samples (*n* = 3) form a distinct cluster along PC2, driven by aliphatic signals tentatively attributed to terpenoids (Figure 4b). Nearly all Australian propolis samples (blue, *n* = 5) are clustered to the left along the PC1 axis, primarily due to a higher beeswax content (see loadings), consistent with the wax analysis. Scandinavian propolis, colored by country to emphasize similarity, span the whole PC1 axis, with negative scores associated with high beeswax content like the Australians and positive scores primarily associated with high aromatic signals.

In the scores plot of the hydrophilic extract (Figure 4c), most *A. mellifera* samples are grouped together in the middle of the scores plot, but with the majority of the Australian propolis samples diverging from Scandinavian samples along PC1. Notably, two Australian samples grouped with the Scandinavian cluster, whereas one Danish and one Norwegian sample deviated markedly from their main group. The cerumen samples of *T. carbonaria* (*n* = 3) form a separate group along PC2. According to the loadings plot (Figure 4d), PC1 separation was primarily influenced by signals at chemical shifts of 3.0–5.5 ppm, attributed to carbohydrates (negative loadings) and 6.0–8.0 ppm, attributed to aromatic compounds, constituted by mainly polyphenols (positive loadings). This suggests a general trend of higher carbohydrate content in Australian propolis and greater aromatic content in Scandinavian propolis. Additionally, cerumen separation along PC2 appeared linked to differences in carbohydrate composition, as indicated by loadings driven by specific carbohydrate signals. In the PC2 loadings plot, anomeric signals at 4.65 ppm (d, J = 7.9 Hz) and 4.60 ppm (d, J = 7.9 Hz) contribute to the positive and negative driving of the loadings, respectively, indicating a distinct carbohydrate profile for cerumen samples.

Overall, this PCA analysis underscores significant compositional differences in propolis and cerumen across both species and geographical regions. Scandinavian propolis generally contained higher levels of aromatic compounds, while Australian propolis was richer in carbohydrates. Cerumen samples, in turn, were characterized by a prominent aliphatic profile, tentatively attributed to terpenoid‐like constituents, along with a distinct carbohydrate profile.

### 3.4 Identification of Spectral Signals Important to RSA Using *r*PLS Regression

Following the analysis of overall chemical differences across extracts, the focus shifted toward identifying polar metabolites associated with RSA. To this end, previously reported RSA data [44] (Table 1) were combined with the present ^1^H NMR data to construct an *r*PLS regression model. Australian samples were excluded from the dataset due to their distinct spectral profiles. Only the hydrophilic fraction was modeled, as extensive aromatic signal overlap in the hydrophobic spectra precluded reliable variable attribution and subsequent structure elucidation. For the sake of completeness and transparency, the development of variable weights in *r*PLS modelling of the hydrophobic data has been included in the Supporting Information (Figure S3).

**TABLE 1:** DPPH RSA of propolis from *A. mellifera* in Australia (*n* = 5) and Scandinavia (*n* = 41) and cerumen from *T. carbonaria* in Australia (*n* = 3), expressed relative to the blank [44].

	*A. mellifera* (SC)	*A. mellifera* (AU)	*T. carbonaria* (AU)
DPPH RSA (%) (mean ± SD)	42.6 ± 12.8	7.6 ± 10.2	18.1 ± 3.2

**FIGURE 5:**
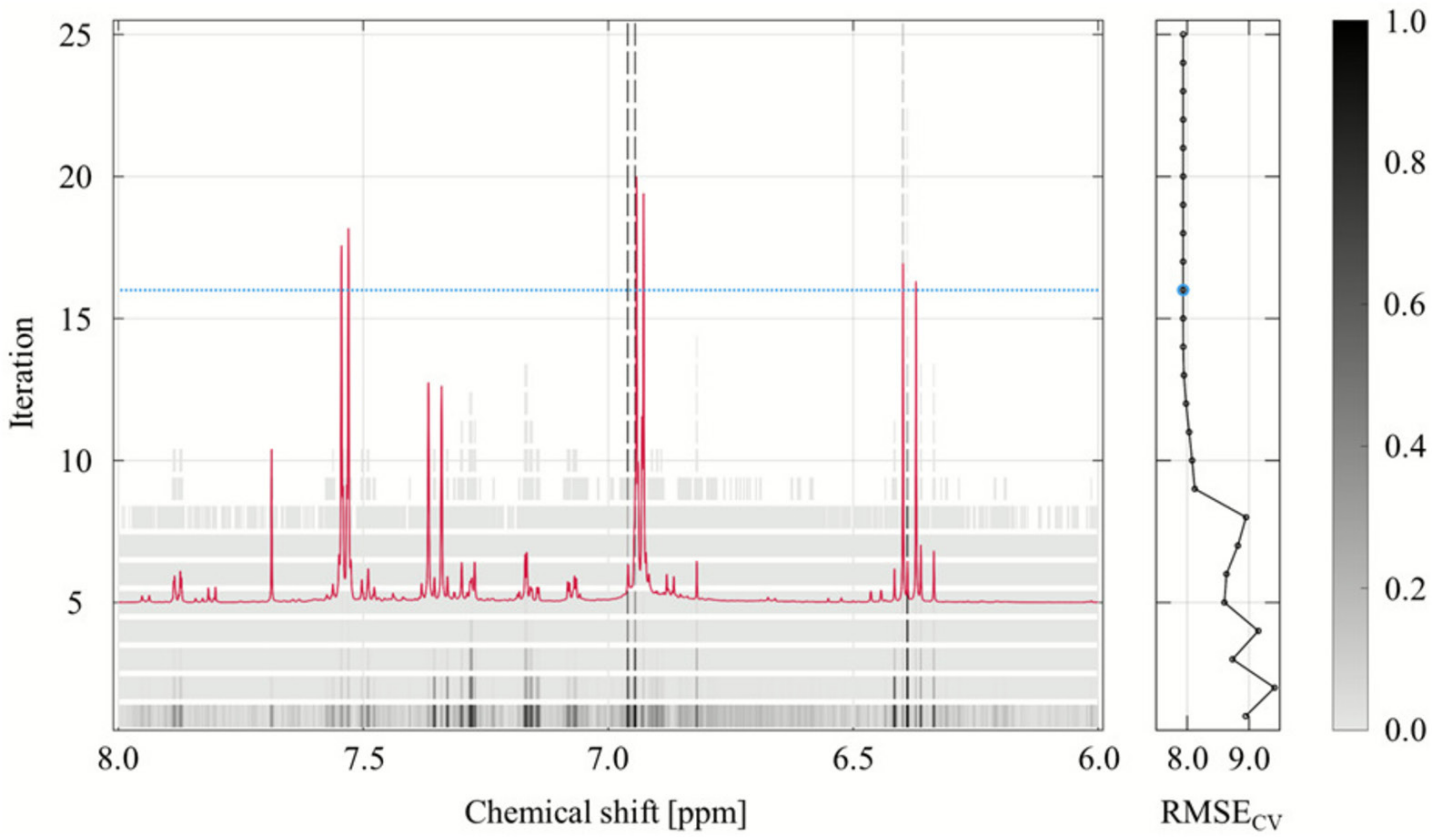
Development of variable weights across recursive iterations in the three latent variable *r*PLS model. The grayscale lines in the main panel represent the evolution of normalized weights for each spectral variable over 25 iterations, illustrating how variable influence changes through recursive weighting. The red line overlays the average ^1^H NMR spectrum (6.0–8.0 ppm) of the hydrophilic phase extracts for reference. The blue stippled horizontal line marks the iteration with the lowest RMSE_CV_; as RMSE_CV_ plateaued beyond this point, this iteration was employed as final *r*PLS model. The right‐side plot shows the RMSE_CV_ at each iteration, confirming model stability across the recursive process. The scale indicates normalized weights, where a value of 1 corresponds to high importance in the model.

Thus, *r*PLS was applied to Pareto‐scaled ^1^H NMR spectra against RSA data, with variable weights recursively reweighted across 25 iterations without enforced variable pruning. The evolution of variable weights and corresponding RMSE_CV_ is shown in Figure 5.

Early iterations (1–8) exhibited unstable RMSE_CV_ accompanied by gradual reduction in variable count. A pronounced reduction in both RMSE_CV_ and variable count occurred between iterations 8 and 9, after which RSME_CV_ gradually declined until iteration 16, where it reached its minimum at eight selected variables. Beyond this point, RMSE_CV_ plateaued while the number of variables continued to drop until reaching three variables, consistent with the number of latent variables used. Iteration 16 was therefore selected as the final model, balancing predictive performance and retention of relevant signals.

**FIGURE 6:**
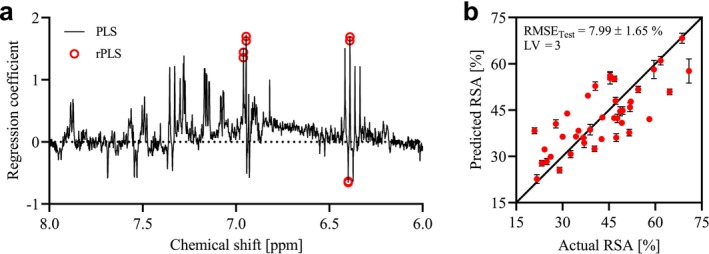
(a) Regression coefficients from full PLS regression (three latent variables) based on Pareto‐scaled ^1^H NMR data. Variables selected by the *r*PLS model are highlighted as red circles. (b) Actual versus predicted RSA for the test sets of the MCCV, using the *r*PLS model (three latent variables). Data are shown as mean ± SD.

Figure 6a displays the regression coefficients from a full PLS model (iteration 1), calculated using the same cross‐validation strategy and number of latent variables as the *r*PLS model, with the *r*PLS‐selected variables highlighted.

These variables, predominantly positively correlated with RSA, clustered into four distinct peaks at 6.390, 6.399, 6.945, and 6.960 ppm, pinpointing the spectral regions most relevant for activity. To test the robustness of these selected variables, MCCV (1000 repetitions) was performed. The predictive performance of the *r*PLS model is shown in Figure 6b as actual versus predicted RSA plots, yielding an RMSE_test_ of 7.99% ± 1.65%. Given the solid robustness of the *r*PLS model, structural elucidation of the selected signals was pursued using STOCSY.

### 3.5 Identification of Compounds Correlated to RSA Using STOCSY

**FIGURE 7:**
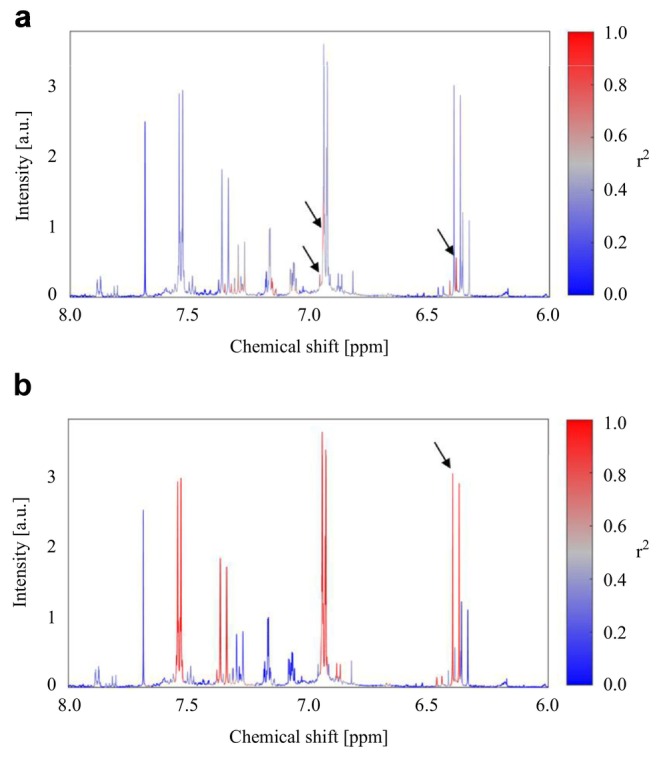
STOCSY analysis leading to the identification of *p*‐coumaric and ferulic acid using *r*PLS selected variables as driver signals. Black arrows indicate signals highlighted by *r*PLS. (a) STOCSY plot using the signal at 6.390 ppm as driver. (b) STOCSY plot using the signal at 6.399 ppm as driver. Signals are colored according to their squared correlation.

To identify the compounds corresponding to the *r*PLS‐highlighted signals, STOCSY was applied using the selected variables as driver signals (Figure 7). Using the variable at 6.390 ppm as driver, several highly correlated signals were revealed, including signals at 6.960 ppm (d, J = 8.16 Hz), 7.152 ppm (dd, J = 8.16, 1.90 Hz), and 7.340 ppm (d, J = 15.94 Hz). The signal at 6.390 ppm appeared as a doublet with a coupling constant of 15.94 Hz, thus displaying coupling patterns characteristic of 3,4‐substituted cinnamic acid derivatives such as caffeic and ferulic acid. However, it could not be ruled out that the signal‐rich region between 3.0 and 4.0 ppm obscured a singlet at approximately 3.9 ppm, which is typical of the methoxy group in ferulic acid, and STOCSY was therefore unable to conclusively distinguish between the two compounds. Standard addition experiments confirmed that *r*PLS selected ferulic acid, revealing that the methoxy singlet was indeed masked by overlapping signals (Table 2).

**TABLE 2:** Chemical shifts (ppm), J‐coupling (Hz), and multiplicity of signals identified by STOCSY and standard addition.

Compound	Chemical shift (ppm) (multiplicity and J‐coupling [Hz])
Ferulic acid	3.915 (s)^b^, 6.390^a^ (d, J = 15.94 Hz), 6.945^a^ (d, J = 8.16), 7.152 (dd, J = 8.16, 1.90 Hz), 7.281 (d, J = 1.91 Hz)^b^, 7.340 (d, J = 15.94 Hz)
*p*‐Coumaric acid	6.399^a^ (d, J = 16.0 Hz), 6.934 (d, J = 8.60 Hz), 7.357 (d, J = 15.97 Hz), 7.537 (d, J = 8.60 Hz)

*Note:* Signals characteristic of ferulic acid and *p*‐coumaric acid are shown with their respective splitting patterns. Keys: s: singlet; d: doublet; dd: double doublet.

^a^ Highlighted by *r*PLS.

^b^ Could only be identified after standard addition.

Similarly, using the 6.399 ppm peak (d, J = 16.0 Hz) as driver revealed highly correlated signals at 6.934 ppm (d, J = 8.60 Hz), 7.357 ppm (d, J = 15.97 Hz), and 7.537 ppm (d, J = 8.60 Hz). These chemical shifts and coupling patterns are very characteristic of *p*‐coumaric acid, and this was confirmed by standard addition.

### 3.6 Quantitative Comparison of Ferulic and *p*‐Coumaric Acid Across Origin and Bee Species

To enable a direct comparison of ferulic and *p*‐coumaric acid levels across different origins and bee species, these compounds were quantified from the ^1^H NMR spectra of the hydrophilic extracts of propolis and cerumen.

**FIGURE 8:**
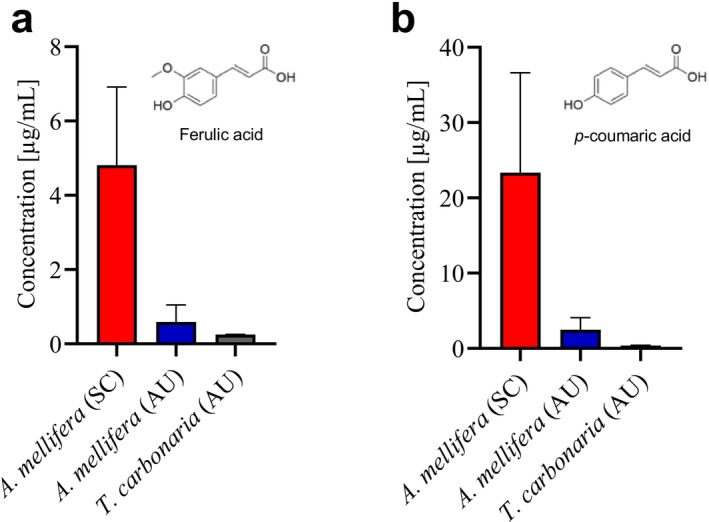
Bar plots showing the quantitative comparison of (a) ferulic acid and (b) *p*‐coumaric acid in hydrophilic extracts of propolis from Scandinavia (SC, *n* = 41) and Australia (AU, *n* = 5) *A. mellifera* and cerumen from *T. carbonaria* (*n* = 3). Bars and vertical lines indicate mean + standard deviation (SD).

Despite large variation, the quantitative NMR analysis revealed substantially higher levels of both ferulic acid and *p*‐coumaric acid in Scandinavian *A. mellifera* propolis compared to Australian samples (Figure 8). Australian *A. mellifera* propolis contained lower amounts of these compounds, while cerumen from *T. carbonaria* exhibited only trace levels, if detected at all. Notably, in both Scandinavian and Australian propolis, average *p*‐coumaric acid concentrations were considerably higher than those of ferulic acid.

## 4 Discussion

### 4.1 Resin and Beeswax Are Origin‐ and Species‐Specific

PCA highlighted clear differences associated with both geographic origin and bee species. Notably, cerumen samples exhibited a markedly more aliphatic profile than propolis, tentatively assigned to terpenoids. Although the ^1^H NMR‐based assignment of these aliphatic signals remains tentative, the pattern is consistent with previous analyses showing that *T. carbonaria* cerumen is rich in terpenoids such as pimaric and abietic acids, β‐amyrin, and multiple sterols [45]. The presence of such compounds is also consistent with the documented resin‐foraging behavior of stingless bees, which rely on olfactory cues from terpenes and preferentially collect terpenoid‐rich resins [46]. These terpenoids contribute to nest defense through the release of antimicrobial volatiles [47], an effect further amplified by the elevated nest temperatures typical of tropical and subtropical climates where these bees thrive. In contrast, *A. mellifera* , native to cooler regions, produced propolis with a lower relative proportion of terpenoids and a higher content of phenolic compounds, reflecting different foraging strategies and a reliance on less volatile chemical defenses. The low levels of aromatic compounds in Australian propolis may be due to the limited availability of poplar trees (e.g., *Populus nigra* ) [48], favored by *A. mellifera* , which are not native to Australia, thereby compelling bees to utilize resins from less phenolic‐rich plant sources. The differences in resins available for foraging could also explain the increased contents of carbohydrates in both Australian propolis and cerumen. Furthermore, the seemingly different carbohydrate profile observed between Australian propolis and cerumen highlights differences in resins foraging. Interestingly, our previous vibrational spectroscopy study on this same sample set [44] proposed that the Australian samples differed from the Scandinavian ones due to variations in carbohydrate and/or terpenoid content. The present results confirm this result, demonstrating that Australian propolis contained higher levels of carbohydrates, while cerumen samples exhibited elevated levels of both carbohydrates and terpenoids.

Both PCA and subsequent beeswax analysis highlighted differences in wax content between propolis and cerumen. The higher wax content observed in Australian propolis likely reflects both limited access to preferred resin‐producing flora and the influence of higher temperatures, as resins are softer and more fluid in warmer climates. Incorporating additional wax may thus enhance stiffness and structural integrity, helping bees maintain the necessary consistency and cohesion of propolis. In contrast, Scandinavian bees live in cooler climates and have abundant access to poplar resins, resulting in propolis with a higher resin‐to‐wax ratio and richer phenolic composition. This hypothesis also helps explain why *T. carbonaria* cerumen contains even less wax, as stingless bees collect large quantities of resin used not only for antimicrobial defense but also as a primary structural material in nest construction, reducing reliance on wax as a binder [49].

While the beeswax content in propolis varied with geographic origin, the wax structure of *A. mellifera* , assessed by chain length as well as degrees of esterification and acidification, remained relatively consistent. In contrast, *T. carbonaria* wax exhibited shorter chain lengths, a lower degree of esterification, and a higher proportion of free fatty acids. The shorter wax chains observed in stingless bees have previously been described [50] and may be attributed to their nesting behavior, where increased flexibility is advantageous for the construction and maintenance of nest structures. The higher proportion of free fatty acids relative to esters in *T. carbonaria* may similarly enhance antimicrobial protection within their resin‐rich nests, although this functional role remains speculative.

### 4.2 *r*PLS Identified Important Metabolites Linked RSA in the Hydrophilic Extracts of Propolis

*r*PLS regression was employed to model the relationship between signals in the aromatic region of the hydrophilic phase ^1^H NMR spectra and RSA. Based on MCCV, the *r*PLS model yielded a mean RMSE_test_ of 8.0% relative to a mean RSA of 42.6%, which indicates only moderate predictive strength. This outcome is not unexpected given that the analysis was limited to the hydrophilic phase, as substantial aromatic overlap in the hydrophobic phase hindered precise variable identification by *r*PLS, while many compounds with radical scavenging potential, such as flavonoids, were likely concentrated in the hydrophobic phase and were therefore not captured by the model. Despite the moderate overall predictive strength, the *r*PLS highlighted signals attributable to ferulic acid as positively associated with RSA, suggesting that this compound serves as an important marker of anti‐oxidative capacity within these hydrophilic extracts. This interpretation is further supported by literature demonstrating that hydroxycinnamic acids bearing multiple electron‐donating groups, such as the methoxy‐hydroxy substitution in ferulic acid, enhance radical stabilization [51]. In contrast, signals from *p*‐coumaric acid were negatively associated with RSA in the PLS models, consistent with lower intrinsic antioxidant potency reported in literature [51], due to its structure bearing only a single para‐positioned hydroxyl group, offering less effective radical stabilization. Although ferulic acid was detected in only modest amounts in the hydrophilic phase, its methoxy substitution may favor partitioning into the hydrophobic fraction, which may partially explain its lower representation in the hydrophilic phase. Nonetheless, the identification of ferulic acid signals despite these concentrations suggests that it contributes to the antioxidant activity of propolis, while also serving as a useful marker for compounds or pathways associated with enhanced radical scavenging activity. These findings also indicate that hydrophilic extracts of propolis can still retain significant antioxidant capacity, suggesting that high ethanol concentrations may not always be necessary for effective extraction of compounds of interest, which could be advantageous in food, cosmetic, or pharmaceutical formulations for pediatric use where ethanol is undesirable. Furthermore, these results demonstrate the utility of *r*PLS in spectroscopic analysis of complex mixtures by effectively pinpointing relevant variables through recursive weighting.

In line with the PCA results, Scandinavian propolis contained considerably higher levels of both ferulic acid and *p*‐coumaric acid compared to Australian propolis and cerumen. Within Scandinavia, Danish and Norwegian samples showed broadly similar concentrations, although *p*‐coumaric acid appeared slightly lower on average in the Norwegian samples. As several Danish samples overlapped with the Norwegian range and the Norwegian group was smaller, this apparent difference was deemed too weak to draw firm conclusions. Consistently, Danish and Norwegian propolis exhibited comparable RSA, and both showed substantially higher antioxidant activity than the Australian sample [44]. Notably, within the hydrophilic extracts of propolis, *p*‐coumaric acid was present at higher concentrations than ferulic acid. However, given the greater lipophilicity of ferulic acid and the resulting tendency to partition into the hydrophobic phase, it remains unclear whether *p*‐coumaric acid is truly more abundant overall. Nevertheless, since the partitioning behavior would be consistent across samples, these observations support the broader conclusion from the PCA that resins collected by Scandinavian *A. mellifera* are generally richer in aromatic compounds, including both ferulic and *p*‐coumaric acids, with ferulic acid emerging as a key contributor to the hydrophilic extractable antioxidant capacity of propolis.

## 5 Conclusions

This study demonstrates the advantages of ^1^H NMR coupled with advanced multivariate data analysis for detailed characterization of propolis and cerumen and for identifying metabolites associated with RSA. Geographic origin and bee species strongly influenced chemical composition, with Scandinavian propolis showing higher levels of aromatic constituents compared to Australian propolis and stingless bee cerumen. The application of *r*PLS, combined with STOCSY, enabled the identification of specific RSA‐related signals, highlighting ferulic acid as an important marker within the hydrophilic extracts. Hydrophilic and hydrophobic extracts displayed clear chemical differences, underscoring the impact of extraction liquid choice in which bioactive constituents are captured.

Overall, these findings emphasize the complementarity of spectroscopic approaches for chemotyping and bioactivity assessment and the importance of extraction strategy and provenance in shaping the chemical and functional diversity of propolis.

## Author Contributions

All authors contributed to the conception and design of the study. Material preparation, data collection, and data analysis were performed by Jonas Pordel Vind, Violetta Aru, Knud Josefsen, and Søren Balling Engelsen. Jonas Pordel Vind prepared the first draft of the manuscript. All authors contributed to manuscript review and editing and approved the final version of the manuscript.

## Conflicts of Interest

The authors declare no conflicts of interest.

## Supporting information


**Figure S1:** Correlation between IR absorbance signals at 1710 cm^−1^ (fatty acids) and 1736 cm^−1^ (fatty esters) to the ^1^H NMR region of interest containing the multiplet (2.15–2.25 ppm). ^1^H NMR signals were aligned with *i*coshift [1].
**Figure S2:** mrc70082‐sup‐0001‐Supporting_Information.docx. ^1^H NMR and IR region of interest in relation to fatty esters and fatty acids.
**Figure S3:** Development of variable weights across recursive iterations in the three component *r*PLS model. ^1^H NMR data were Pareto‐scaled, while RSA data were mean‐centered. The grayscale lines in the main panel represent the evolution of normalized weights for each spectral variable over 25 iterations, illustrating how variable influence changes through recursive weighting. The red line overlays the average ^1^H NMR spectrum (6.0–8.0 ppm) of the hydrophobic phase extracts for reference. The blue stippled horizontal line marks the iteration with the lowest RMSE_CV._ The right‐side plot shows the RMSE_CV_ at each iteration, confirming model stability across the recursive process. The scale indicates normalized weights, where a value of 1 corresponds to high importance in the model.
**Table S1:** Overview of the geographical origin of propolis and cerumen.

## Data Availability

The data that support the findings of this study are available from the corresponding author upon reasonable request.
