# Supplementary material for: Comparative 1H NMR Metabolomics Between Scandinavian Propolis and Australian Propolis: The Quest to Identify Radical Scavenging Compounds
Source: Magn Reson Chem. 2026 Jan 28;64(4):438–50. doi: 10.1002/mrc.70082 (PMC12950336; doi:10.1002/mrc.70082)
Supplement: Supplementary file 1 — Figure S1: Correlation between IR absorbance signals at 1710 cm−1 (fatty acids) and 1736 cm−1 (fatty esters) to the 1H NMR region of interest containing the multiplet (2.15–2.25 ppm). 1H NMR signals were aligned with icoshift [1]. Figure S2: mrc70082‐sup‐0001‐Supporting_Information.docx. 1H NMR and IR region of interest in relation to fatty esters and fatty acids. Figure S3: Development of variable weights across recursive iterations in the three component rPLS model. 1H NMR data were Pareto‐scaled, while RSA data were mean‐centered. The grayscale lines in the main panel represent the evolution of normalized weights for each spectral variable over 25 iterations, illustrating how variable influence changes through recursive weighting. The red line overlays the average 1H NMR spectrum (6.0–8.0 ppm) of the hydrophobic phase extracts for reference. The blue stippled horizontal line marks the iteration with the lowest RMSECV. The right‐side plot shows the RMSECV at each iteration, confirming model stability across the recursive process. The scale indicates normalized weights, where a value of 1 corresponds to high importance in the model. Table S1: Overview of the geographical origin of propolis and cerumen. [file MRC-64-438-s001.docx]

**Supporting information**

**Comparative ^1^H NMR Metabolomics between Scandinavian Propolis and Australian Propolis: The Quest to Identify Radical Scavenging Compounds**

Jonas Vind^1^, Søren Balling Engelsen^2^, Henrik Munch Jørgensen^1^, Julie Christine Antvorskov^3^, Knud Josefsen^1^, Violetta Aru^2^.

Affiliations:

^1^Department of Pathology, The Bartholin Institute, Copenhagen University Hospital, Copenhagen, Denmark

^2^Department of Food Science, University of Copenhagen, Rolighedsvej 26, 1958 Frederiksberg C, Denmark

^3^Department of Clinical Research, Steno Diabetes Center Copenhagen, Translational Type 1 Diabetes Research, Herlev, Denmark

**Table S1.** Overview of the geographical origin of propolis and cerumen

| **Sample ID** | **Geographic location** | | **Species** |
| --- | --- | --- | --- |
|  | **Country** | **Region** |  |
| 1 | Denmark | Region Zealand | *Apis mellifera* |
| 2 | Denmark | Region Zealand | *Apis mellifera* |
| 3 | Denmark | Region Zealand | *Apis mellifera* |
| 4 | Denmark | Region Zealand | *Apis mellifera* |
| 5 | Denmark | Region Zealand | *Apis mellifera* |
| 6 | Denmark | Region Southern Denmark | *Apis mellifera* |
| 7 | Denmark | Region Northern Denmark | *Apis mellifera* |
| 9 | Denmark | The Capital Region | *Apis mellifera* |
| 10 | Denmark | Region Southern Denmark | *Apis mellifera* |
| 11 | Denmark | Region Southern Denmark | *Apis mellifera* |
| 12 | Denmark | Region Southern Denmark | *Apis mellifera* |
| 13 | Denmark | Region Southern Denmark | *Apis mellifera* |
| 14 | Denmark | Central Denmark Region | *Apis mellifera* |
| 15 | Denmark | Central Denmark Region | *Apis mellifera* |
| 16 | Denmark | Region Zealand | *Apis mellifera* |
| 17 | Denmark | Central Denmark Region | *Apis mellifera* |
| 18 | Denmark | Region Southern Denmark | *Apis mellifera* |
| 19 | Denmark | Central Denmark Region | *Apis mellifera* |
| 20 | Denmark | Region Northern Denmark | *Apis mellifera* |
| 21 | Denmark | Region Zealand | *Apis mellifera* |
| 22 | Denmark | Region Southern Denmark | *Apis mellifera* |
| 23 | Denmark | Region Southern Denmark | *Apis mellifera* |
| 24 | Denmark | Region Southern Denmark | *Apis mellifera* |
| 25 | Denmark | Region Northern Denmark | *Apis mellifera* |
| 26 | Denmark | Central Denmark Region | *Apis mellifera* |
| 27 | Denmark | Region Northern Denmark | *Apis mellifera* |
| 28 | Denmark | Region Northern Denmark | *Apis mellifera* |
| 29 | Denmark | Region Zealand | *Apis mellifera* |
| 33 | Denmark | Region Southern Denmark | *Apis mellifera* |
| 34 | Denmark | Region Southern Denmark | *Apis mellifera* |
| 35 | Denmark | Region Zealand | *Apis mellifera* |
| 36 | Denmark | The Capital Region | *Apis mellifera* |
| 37 | Denmark | Region Zealand | *Apis mellifera* |
| 38 | Denmark | Region Northern Denmark | *Apis mellifera* |
| 39 | Denmark | Central Denmark Region | *Apis mellifera* |
| 40 | Denmark | Central Denmark Region | *Apis mellifera* |
| 41 | Norway | Viken | *Apis mellifera* |
| 42 | Norway | Viken | *Apis mellifera* |
| 44 | Norway | Vestland | *Apis mellifera* |
| 45 | Norway | Vestland | *Apis mellifera* |
| 46 | Norway | Agder | *Apis mellifera* |
| 47 | Norway | Agder | *Apis mellifera* |
| 48 | Australia | New South Wales | *Apis mellifera* |
| 49 | Australia | New South Wales | *Apis mellifera* |
| 50 | Australia | New South Wales | *Apis mellifera* |
| 51 | Australia | New South Wales | *Apis mellifera* |
| 52 | Australia | New South Wales | *Apis mellifera* |
| 53 | Australia | New South Wales | *Tetragonula carbonaria* |
| 54 | Australia | New South Wales | *Tetragonula carbonaria* |
| 55 | Australia | New South Wales | *Tetragonula carbonaria* |

**Figure S1.** Correlation between IR absorbance signals at 1710 cm^-1^ (fatty acids) and 1736 cm^-1^ (fatty esters) to the ^1^H NMR region of interest containing the multiplet (2.15–2.25 ppm). ^1^H NMR signals were aligned with *i*coshift ^[1]^.

*A. mellifera* (SC):

*A. mellifera* (AU):

*T. carbonaria* (AU):

**Figure S2.** ^1^H NMR and IR region of interest in relation to fatty esters and fatty acids.


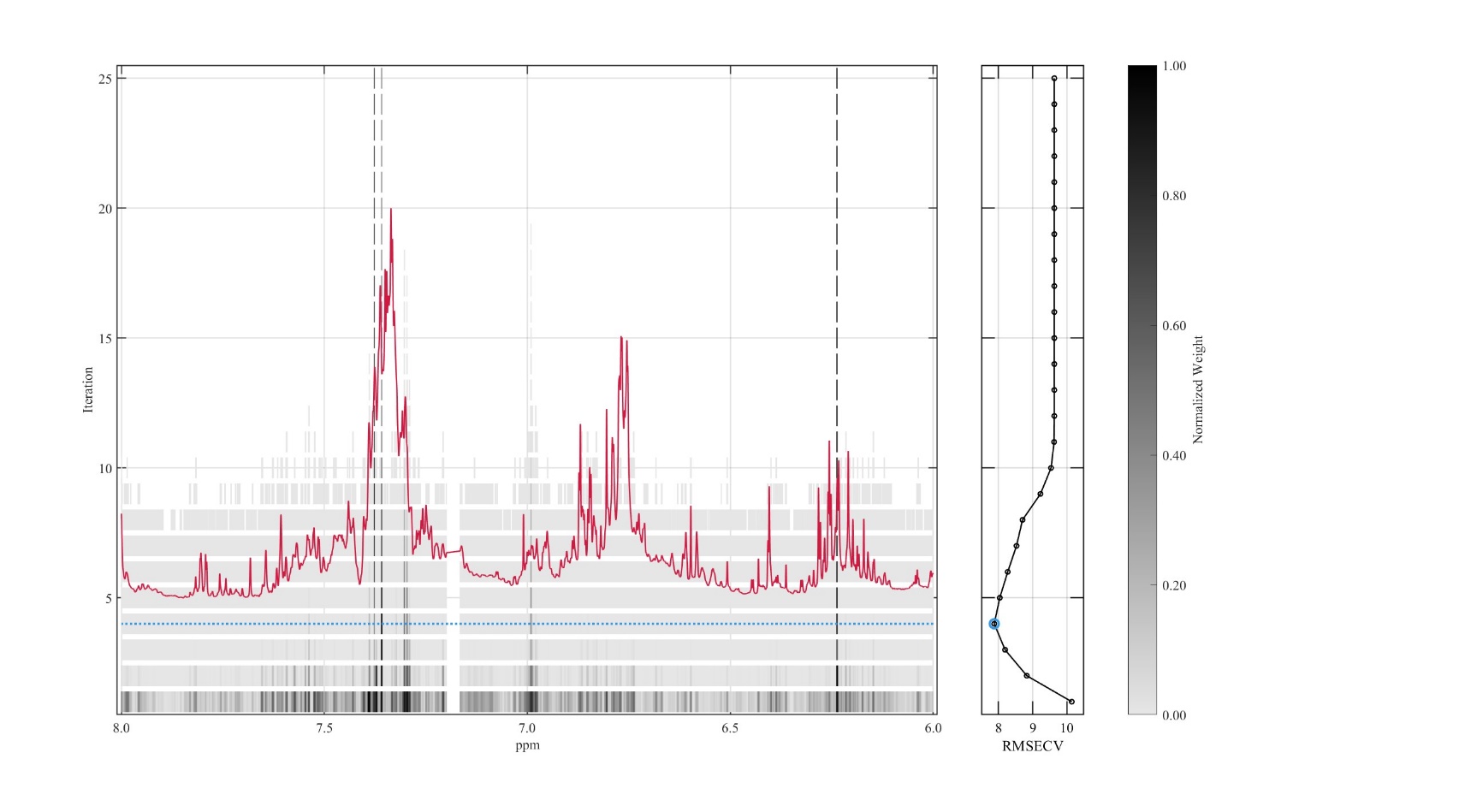


Figure S3 Development of variable weights across recursive iterations in the three component *r*PLS model. ^1^H NMR data was Pareto-scaled, while RSA data was mean-centered. The grayscale lines in the main panel represent the evolution of normalized weights for each spectral variable over 25 iterations, illustrating how variable influence changes through recursive weighting. The red line overlays the average ^1^H NMR spectrum (6.0-8.0 ppm) of the hydrophobic phase extracts for reference. The blue stippled horizontal line marks the iteration with the lowest RMSE_CV._ The right-side plot shows the RMSE_CV_ at each iteration, confirming model stability across the recursive process. The scale indicates normalized weights, where a value of 1 corresponds to high importance in the model.

**References**

[1] F. Savorani, G. Tomasi, S. B. Engelsen, *J Magn Reson* **2010**, *202*, 190-202.
